# Descriptive analysis of autologous and xenograft materials for secondary alveolar bone grafting in cleft lip and palate patients: a literature review

**DOI:** 10.1186/s40902-025-00477-6

**Published:** 2025-08-20

**Authors:** Jihye Ryu, Dae-Seok Hwang

**Affiliations:** Department of Oral and Maxillofacial Surgery, Pusan ​​National University Dental Hospital, Yangsan, Korea, Republic of

**Keywords:** Xenograft, Autogenous, Cleft, Secondary alveolar bone graft

## Abstract

**Background:**

This study aims to evaluate the outcomes of secondary alveolar bone grafting in patients with cleft lip and palate by comparing the clinical effectiveness of autologous bone grafts and xenogeneic graft materials. The objective is to provide evidence-based insight into the comparative efficacy of these grafting approaches.

**Main body:**

A systematic search of the literature published between January 2004 and January 2023 identified 606 studies. Following a series of screening processes, five studies met the inclusion criteria, including only two randomized controlled trials (RCTs). The selected studies specifically evaluated xenogeneic bone graft materials derived from bovine sources, with allograft materials excluded. The results demonstrated no statistically significant difference between autologous and xenograft grafts in terms of postoperative bone graft volume and height in patients with cleft lip and palate. These findings provide important insight into the comparative effectiveness of grafting materials used in secondary alveolar bone grafting.

**Conclusions:**

In summary, the findings indicate that autologous and xenogeneic graft materials yield comparable outcomes in secondary alveolar bone grafting for patients with cleft lip and palate. These results may inform clinical decision-making and guide future research in optimizing grafting strategies.

## Background

Secondary alveolar bone grafting (SABG) is a critical component of comprehensive rehabilitation in patients with cleft lip and palate. This procedure not only restores the continuity of the maxillary arch but also supports the nasal base, facilitates orthodontic movement, permits eruption of adjacent teeth into the cleft site, and provides sufficient bone volume for future dental implant placement. Various grafting materials—both autologous and synthetic—have been employed to manage alveolar cleft defects [1].

Autologous bone grafts, particularly those harvested from the iliac crest, remain the gold standard due to their inherent osteogenic properties and the presence of osteoinductive and osteoconductive properties [2]. However, autogenous grafting requires a secondary surgical site, contributing to increased patient morbidity, extended operative time, and longer hospital stays. In an effort to mitigate these drawbacks, xenogeneic grafts—especially those derived from bovine sources—have emerged as a promising alternative [2–7].

Despite the increasing clinical use of xenografts, particularly bovine bone substitutes, there is a notable lack of comparative studies directly evaluating the outcomes of autologous versus xenogeneic bone grafts in secondary alveolar bone grafting. The aim of this study is to perform a descriptive analysis and conduct a literature review of secondary alveolar bone grafting using autologous versus xenograft material in patients with cleft lip and palate.

## Methods

A comprehensive literature search was conducted across PubMed, Embase, and the Cochrane Library using the keywords “cleft,” “lip,” “palate,” “secondary,” “alveolar,” and “bone graft” to identify studies relevant to alveolar bone reconstruction in patients with cleft lip and palate. The initial search yielded 606 articles. After the removal of 310 duplicates, 296 articles were subjected to full-text review. Each study was carefully assessed for relevance based on the inclusion criteria focusing on the clinical use of xenogeneic bone graft materials.

**Fig. 1:**
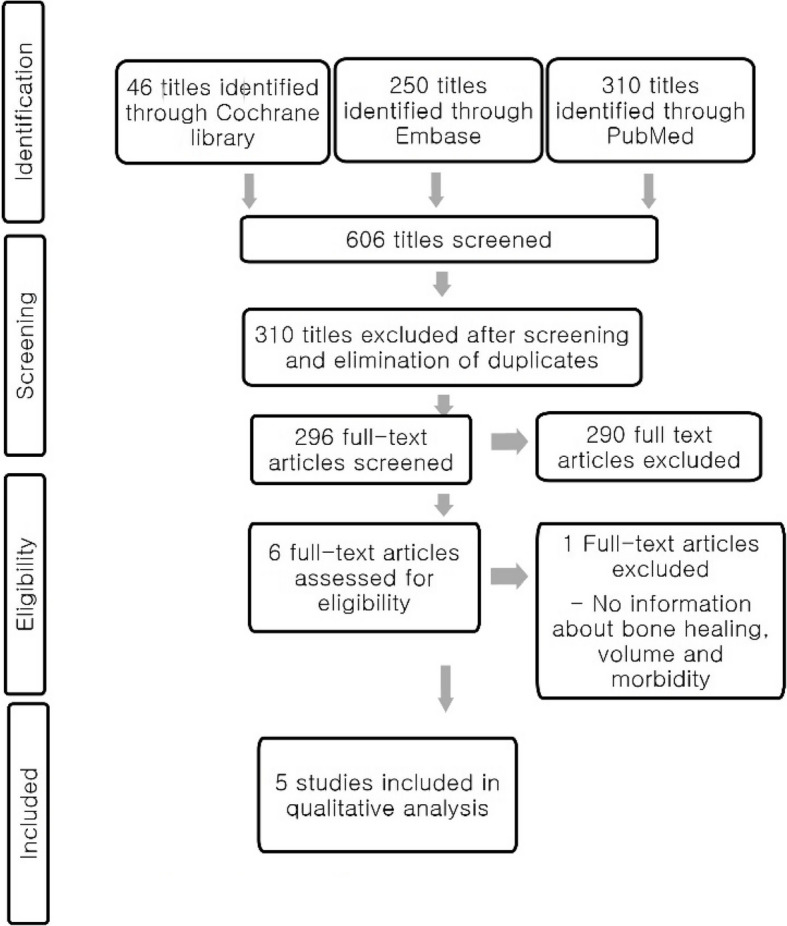
Flow diagram of the review

Of the 296 full-text articles evaluated, 290 were excluded for not meeting the inclusion criteria—most commonly due to a lack of focus on xenograft materials. This yielded a final selection of six studies. One of these was excluded due to the combined use of xenograft with autologous bone, which fell outside the defined scope of the study. Ultimately, five studies were included in the final analysis, among which only two qualified as a randomized controlled trial (RCT)(Fig. 1).

All included studies investigated xenogeneic bone graft materials, primarily derived from bovine sources. Studies involving allograft materials were deliberately excluded. While there is an increasing body of literature comparing synthetic and autologous graft materials for cleft repair, inconsistencies in graft material composition, study design, and outcome measures precluded the feasibility of conducting a systematic review or meta-analysis. As such, this study adopts a descriptive review approach, summarizing the findings of the five selected papers to provide insight into the comparative effectiveness of xenogeneic versus autologous grafting techniques in secondary alveolar bone grafting.

## Results

A total of five clinical studies comparing bovine-derived xenografts to autogenous bone in SABG were reviewed. The studies demonstrated heterogeneity in design, graft source, sample size, patient age, and outcome assessment. Four studies (Bahtiar et al., Alnajjar et al., Benlidayi et al., and Kumar et al.) used iliac crest bone as the control graft, while one (Bezerra et al.) used mandibular symphysis. Study designs included two retrospective studies (Bahtiar, Benlidayi), one pilot study (Bezerra), and two prospective randomized controlled trials (Alnajjar and Kumar) with sample sizes ranging from 15 to 23 patients. Age distributions varied significantly: Bahtiar’s cohort focused on early childhood (3–6 years), while the others included older children and adolescents, with mean ages ranging from approximately 9.8 to 15.6 years.

Each study also employed different radiographic assessments. Bahtiar and Benlidayi used panoramic radiographs (Bergland and Chelsea scales, respectively) supplemented by cone-beam computed tomography (CBCT) in the latter, while Alnajjar, Bezerra, and Kumar relied exclusively on CBCT to measure bone density (Hounsfield units), area, or volume. Notably, adjunctive biological enhancers such as platelet-rich fibrin (PRF) and platelet-rich plasma (PRP) were used in the studies by Alnajjar and Bezerra, respectively, potentially influencing regenerative outcomes.

Specifically, Bahtiar et al. conducted a retrospective cohort study evaluating bone volume using panoramic radiographs and the Bergland scale in a pediatric population aged 3–6 years. Additionally, in their analysis based on cleft type, 80% of patients had complete clefts, and 20% had incomplete clefts. The distribution of cleft type between the iliac crest bone graft and bovine bone graft groups showed no significant difference (*p* = 0.678) [8]. Alnajjar et al. conducted a study incorporating PRF into the grafting procedure and assessed bone density using CBCT. At the 6-month postoperative follow-up, no statistically significant difference in bone density was observed between groups (*p* = 0.165). Benlidayi et al. analysis combined Chelsea scale bone volume assessment and CBCT-based density measurements, reporting *p*-values of 0.478 and 0.190, respectively, between groups. Kumar V. et al. compared bovine-derived demineralized bone matrix (DMBM) with iliac crest graft over the long term for SABG in patients with unilateral cleft lip and palate using CBCT to calculate bone volume difference and clinical outcomes. The mean volume change using the Cavalieri principle and vertical and horizontal bone height formation did not significantly differ between groups either. This study differed from the others included in the review by employing a longer follow-up period, with a mean duration of 63 months. Bezerra et al. study comparing bovine xenograft with autologous symphyseal grafts was supplemented by platelet-rich plasma (PRP). The study measured graft area and volume using CBCT, with no significant differences (*p* = 0.17 for area; *p* = 0.12 for volume). Collectively, these studies suggest that bovine xenografts perform comparably to autogenous bone grafts in terms of bone volume, density, and integration, though the absence of statistically significant results across all studies highlights the need for further high-quality, randomized trials (Tables 1 and 2).

**Table 1:** Summary of inclusion studies

	**Study 1**	**Study 2**	**Study 3**	**Study 4**	**Study 5**
Authors	Bahtiar, R. et al	Alnajjar, A. et al	Benlidayi, M. E. et al	Bezerra, B. T. et al	Kumar, V. et al
Year published	2018	2020	2012	2019	2021
Study type	Retrospective cohort	Prospective RCT	Retrospective	Pilot	Prospective RCT
Source	C: Iliac crest T: Bovine	C: Iliac crest T: Bovine	C: Iliac crest T: Bovine	C: Symphysis T: Bovine	C: Iliac crest T: Bovine
Application	-	PRF	-	PRP	-
Sample size	15	20	23	20	20
Measurement	Panoramic bone volume by Bergland scale (%)	CBCT bone density (HU)	Bone volume by Chelsea scale (%) and CBCT bone density (HU)	CBCT area (mm^2^) and volume (mm^3^)	CBCT volume (mm^3^) by Cavalieri principle
Mean age (years)	C: 3–6 years (100%) T: 3–6 years (55.6%) > 6 years (44.4%)	C: 10.5 T: 9.8	C: 13 (3.76) T: 10.82 (2.6)	C: 15.6 (3.64) T: 14.5 (7.44)	C: 12 ± 3.23 T: 12.20 ± 2.74
Significance (statistical significance was determined at *P* .05)	*P* = 0.678	*P* = 0.165	*P* = 0.478 (volume) *P* = 0.190 (density)	*P* = 0.17 (area) *P* = 0.12 (volume)	*P* = 0.975 (volume at 14th day) *P* = 0.907 (volume at 6 months)

**Table 2:** A risk-of-bias summary table, following the Cochrane risk-of-bias tool format, with + , ?, and—symbols indicating low, unclear, and high risk respectively for bias domains across five included studies

Study	Random sequence generation (selection bias)	Allocation concealment (selection bias)	Blinding of participants and personnel (performance bias)	Blinding of outcome assessment (detection bias)	Incomplete outcome data (attrition bias)	Selective reporting (reporting bias)	Other bias
Bahtiar et al. (2018)	? (unclear, retrospective)	? (unclear)	- (no blinding)	? (unclear)	? (unclear)	? (unclear)	? (unclear follow-up info)
Alnajjar et al. (2020)	+ (RCT with randomization)	+ (randomly allocated)	+ (blinded)	? (blinding unclear)	? (follow-up 6 months, not fully detailed)	? (unclear)	? (no other biases noted)
Benlidayi et al. (2012)	? (retrospective)	? (unclear)	- (no blinding)	? (unclear)	? (unclear)	? (unclear)	? (unclear follow-up info)
Bezerra et al. (2019)	? (pilot, randomization unclear)	? (unclear)	- (no blinding)	- (allocated prior to detection)	? (unclear)	? (unclear)	? (small sample size)
Kumar et al. (2021)	+ (prospective RCT)	+ (randomly allocated)	+ (blinded)	+ (blinded)	+ (long follow-up, adequate data)	? (unclear)	? (no other biases noted)

Risk of bias was assessed for all five included studies using the Cochrane Collaboration’s risk-of-bias tool. The assessment covered seven domains: random sequence generation, allocation concealment, blinding of participants and personnel, blinding of outcome assessment, incomplete outcome data, selective reporting, and other potential sources of bias. Overall, only two of the five studies (Alnajjar et al. and Kumar et al.) were randomized controlled trials with structured methodologies. Among these, Kumar et al. demonstrated the most rigorous study design and lowest risk of bias. In contrast, the retrospective and pilot studies exhibited methodological weaknesses, including the absence of blinding, limited follow-up, and incomplete reporting, leading to moderate to high risk of bias across several domains.

## Discussion

Recent research in secondary alveolar bone grafting for patients with cleft lip and palate has shown growing interest in xenogeneic graft materials, particularly those derived from bovine sources, as potential alternatives to autologous bone [4, 9, 10]. This shift is largely driven by the advantages of xenografts, including greater availability, ease of handling, and the elimination of donor site morbidity [8, 11]. However, autogenous bone remains the most widely used and well-established material for the reconstruction of bone defects. It is considered the gold standard due to its osteoinductive, osteoconductive, and osteogenic properties. With the development of alternative grafting materials, numerous comparative studies have emerged, evaluating the effectiveness of allografts and xenografts relative to autogenous bone in promoting bone healing and regeneration [9, 11–13]. Various grafting materials have been utilized for the reconstruction of alveolar cleft defects, including bioabsorbable hydroxyapatite, *β*-tricalcium phosphate, DMBM, and bone morphogenetic protein-2 (BMP-2). The identification and development of recombinant human BMP-2, in combination with a collagen sponge carrier, have facilitated the clinical use of alloplastic bone grafts even in SABG. A systematic review limited to randomized controlled trials further confirmed the effectiveness of alloplastic materials for SABG in patients with cleft lip and palate [11]. Among these, bovine-derived DMBM—a resorbable cortical xenograft composed primarily of type I collagen—has gained attention due to its osteoinductive properties, biocompatibility, and safety [5, 7]. Although xenograft material such as Bio-Oss® has been widely studied and applied in various clinical procedures—including periodontal bone defects, post-extraction sockets, dehiscence and fenestration defects, bone cavity filling, and sinus lift surgeries—its clinical use in cleft patients has been limited. This caution likely arises from concerns regarding potential complications specific to the cleft site. Despite its broader clinical utility, the application of bovine-derived xenograft materials in secondary alveolar bone grafting (SABG) remains relatively underexplored. Most existing studies, such as that by Francis et al., have reported outcomes over short-term periods ranging from 6 months to 1 year. However, there remains a notable lack of long-term prospective research evaluating critical parameters such as bone volume maintenance and graft resorption over time, as emphasized by Stasiak et al. and Kumar et al. [5, 6, 14]

These studies have investigated parameters such as bone volume augmentation, graft integration, periodontal outcomes, and long-term stability. Although high-level evidence remains limited, early findings suggest that bovine-derived xenografts may offer comparable outcomes to autologous grafts in secondary alveolar bone grafting [1–3, 8].

### Bone volume and density outcomes

The five reviewed studies consistently reported no statistically significant differences between autogenous and xenogeneic grafts in terms of bone graft volume, density, or height in patients with cleft lip and palate. However, the limited number of direct comparative studies and the considerable methodological variability across the literature constrain definitive conclusions. Bahtiar et al. evaluated graft success using panoramic radiographs and the Bergland scale to quantify alveolar defect closure. Alnajjar et al. used CBCT to assess bone density, with two examiners evaluating grayscale values that were quantitatively converted into Hounsfield units (HU). The study compared autogenous bone chips with bovine bone particles combined with injectable platelet-rich fibrin (I-PRF). Benlidayi et al. utilized panoramic and occlusal radiographs alongside CBCT-based density measurements, applying a semiautomated method to outline and quantify the grafted region. Bezerra et al. measured graft area and volume using an automated navigation system and reported outcomes for xenografts mixed with platelet-rich plasma (PRP). Lastly, Kumar et al. assessed bone volume using the Cavalieri principle applied to CBCT volumetric data, reconstructed and sectioned into 1-mm isotropic slices in the axial plane. To evaluate the newly grafted site with greater precision, they extended the follow-up period, as this method of measurement was only applicable while new bone could still be clearly delineated. As a result, a long-term assessment was conducted, with a mean follow-up duration of 5.25 years (63 months). Despite diverse imaging modalities and evaluation protocols, none of the studies identified significant differences in bone regeneration outcomes between graft types.

### Complications and limitations in using xenograft materials

While xenografts offer practical advantages such as reduced donor site morbidity and surgical time, their clinical use in SABG for cleft patients has been approached cautiously due to concerns over graft integration and complication risks. Postoperative follow-up periods varied considerably among the included studies, potentially affecting the reliability and comparability of their outcomes. Additionally, the nature of the cleft defect—whether complete or incomplete—was not consistently stratified in the outcome analyses, despite its likely influence on graft behavior. Among the five studies, only Kumar et al. provided long-term data on bone resorption and graft stability, underscoring a significant gap in longitudinal evidence. Evaluation of the methodological quality using the Cochrane risk-of-bias tool revealed that only two studies—Alnajjar et al. and Kumar et al.—were randomized controlled trials (RCTs). Kumar et al. demonstrated the strongest methodological rigor, incorporating clear randomization, allocation concealment, blinding of participants and assessors, and comprehensive long-term follow-up, indicating low risk of bias across most domains. In contrast, the retrospective or pilot nature of the remaining studies introduced several sources of bias, including lack of blinding, unclear outcome reporting, and small sample sizes, particularly in Bezerra et al., which further compromises internal validity.

### Use of PRP and PRF with xenografts

Recent studies have explored the adjunctive use of platelet concentrates—namely PRP and PRF—with xenogeneic grafts to enhance bone regeneration. A commonly used xenograft material in this context is Bio-Oss® (Geistlich Pharma AG, Wolhusen, Switzerland), a deproteinized bovine mineral matrix with osteoconductive properties. Bio-Oss acts as a tridimensional scaffold supporting the ingrowth of host capillaries and osteoprogenitor cells, thus contributing to new bone formation. Its structure closely resembles human bone, with a natural porous architecture that facilitates revascularization, enhances blood clot stabilization, and promotes the adsorption of endogenous proteins and growth factors [7, 15–17]. It is available in various forms—including blocks and granules—and is biocompatible, with no reported induction of local or systemic immune responses.

The rationale behind combining platelet-rich plasma (PRP) with Bio-Oss® lies in its ability to release significant amounts of growth factors that may accelerate bone graft maturation and improve healing [18]. Alnajjar et al. evaluated injectable PRF (I-PRF) in combination with bovine xenografts and assessed postoperative bone density using CBCT. However, their 6-month follow-up revealed no statistically significant improvement compared to autogenous grafts. Likewise, Bezerra et al. mixed Bio-Oss® with PRP in a pilot study but found no significant differences in bone area or volume relative to controls. These findings suggest that while biologic adjuncts like PRP and PRF have theoretical and experimental support, their clinical effectiveness in the context of secondary alveolar bone grafting (SABG) remains inconclusive. The heterogeneity of preparation protocols, short follow-up periods, and limited sample sizes across current studies hinder definitive conclusions. Therefore, further well-designed clinical trials are warranted to better understand the potential synergistic effects of PRP and PRF in enhancing xenograft-based alveolar reconstruction.

Moreover, the nature of the cleft defect prior to grafting—whether complete or incomplete, or varying in size and volume—was not consistently classified or controlled. This lack of standardization may influence the interpretation of graft performance. Additionally, the literature is still limited in terms of randomized controlled trials and long-term outcome data assessing the safety, efficacy, and integration of xenografts.

Further research with larger sample sizes, standardized imaging protocols, and consistent classification of cleft morphology is essential to validate current findings and guide clinical decision-making. Longitudinal studies with well-defined outcome measures will be particularly important to evaluate the long-term stability and integration of xenogeneic grafts in alveolar cleft reconstruction.

## Conclusion

Studies comparing xenogeneic and autogenous grafts have provided important insights into their efficacy in promoting bone healing and regeneration in alveolar cleft repair. Although existing research underscores the need for further investigation, preliminary evidence suggests that xenograft materials hold significant potential as an alternative for secondary alveolar bone grafting in patients with cleft lip and palate. Continued research is essential to optimize clinical protocols and evaluate long-term outcomes in comparison to autogenous grafts, ultimately contributing critical evidence to secondary alveolar repair for cleft patients.

Author contributions

Jihye Ryu wrote the main manuscript text and prepared a figure and tables. Jihye Ryu and Dae-Seok Hwang designed the study and reviewed the manuscript.

## Data Availability

No datasets were generated or analysed during the current study.

## Abbreviations

SABG: Secondary alveolar bone grafting
RCT: Randomized controlled trial
CBCT: Cone-beam computed tomography
DMBM: Bovine-derived demineralized bone matrix
BMP-2: Bone morphogenetic protein-2
C: Control group
T: Test group
PRF: Platelet-rich fibrin
PRP: Platelet-rich plasma
